# Myocardial bridging, a trigger for Takotsubo syndrome

**DOI:** 10.1007/s12471-018-1142-0

**Published:** 2018-08-09

**Authors:** A. S. Triantafyllis, S. de Ridder, K. Teeuwen, L. C. Otterspoor

**Affiliations:** 1 Heart Center, Department of Interventional Cardiology, Catharina Hospital Eindhoven, Eindhoven, The Netherlands; 2 Department of Cardiology, St. Anna Hospital Geldrop, Geldrop, The Netherlands

A 71-year-old female presented with angina and ST elevation in leads V2–V4 on the electrocardiogram. Coronary angiography excluded stenotic lesions. A wrap-around left anterior descending (LAD) with myocardial bridging in the mid-segment was observed (Fig. 1a, d, arrowheads, Video 1). Intravascular ultrasound demonstrated systolic compression of the mid-LAD with a minimum lumen area of 3.06 mm^2^ (systole) to 5.02 mm^2^ (diastole) and an echolucent region between the bridged segment and epicardial tissue persisting throughout the cardiac cycle (‘half-moon sign’) (b, e, arrows, Video 2) [1]. Left ventriculography revealed mid-apical ballooning (c, f, arrowheads, Video 3), corresponding with the diagnosis of Takotsubo syndrome. High-sensitive troponin-T (normal ≤30 ng/l) was elevated, reaching a peak (590 ng/l) after 12 h. The patient recalled no trigger. At follow-up she was asymptomatic with normal echocardiography (Video 4).

**Fig. 1:**
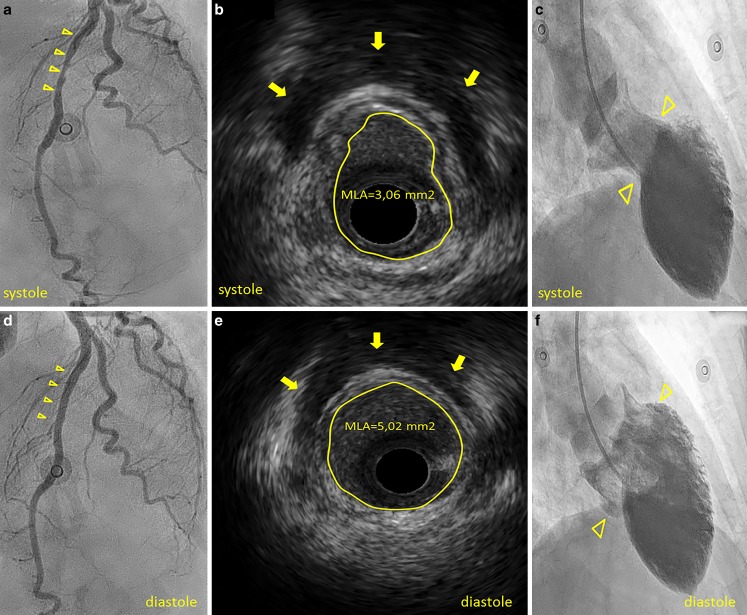
Wrap-around LAD with myocardial bridging in the mid segment (**a** in systole, **d** in diastole, *arrowheads*). Intravascular ultrasound demonstrating systolic compression of the mid-LAD with a mean lumen area oscillating from 3.06 mm^2^ in systole (**b**) to 5.02 mm^2^ in diastole (**e**) and an echolucent region between the bridged coronary segment and epicardial tissue persisting throughout the cardiac cycle, ‘half-moon sign’ (**b, e**, *arrows*). Left ventricular angiography revealing mid-apical ballooning with hypercontractility of the basal segments (**c, f**, *arrowheads*, in systole and diastole respectively)

Myocardial bridging of a wrap-around LAD has been associated with Takotsubo syndrome [2]. Cardiologists should be alert for this presentation given its implication with worse prognosis [3].

## Caption Electronic Supplementary Material

Video 1: Coronary angiography showing myocardial bridging

Video 2: IVUS demonstrating systolic compression of the mid-LAD with a minimum lumen area oscillating from 3.06 mm^2^ in systole to 5.02 mm^2^ in diastole and an echolucent region between the bridged coronary segment and epicardial tissue persisting throughout the cardiac cycle (‘half-moon sign’)

Video 3: Left ventricular (LV) angiography revealing typical mid-apical ballooning with hypercontractility of the basal segments

Video 4: Follow-up Echocardiography illustrating restoration of left ventricular function
